# Bioactive Compounds and Total Sugar Contents of Different Open-Pollinated Beetroot Genotypes Grown Organically

**DOI:** 10.3390/molecules25214884

**Published:** 2020-10-22

**Authors:** Khadijeh Yasaminshirazi, Jens Hartung, Michael Fleck, Simone Graeff-Hoenninger

**Affiliations:** 1 Cropping Systems and Modelling, Institute of Crop Science, University of Hohenheim, Fruwirthstr. 23, 70599 Stuttgart, Germany; simone.graeff@uni-hohenheim.de; 2 Department of Biostatistics, Institute of Crop Science, University of Hohenheim, Fruwirthstr. 23, 70599 Stuttgart, Germany; jens.hartung@uni-hohenheim.de; 3 Kultursaat e.V., Kronstraβe 24, 61209 Echzell, Germany; michael.fleck@kultursaat.org

**Keywords:** beetroot, open-pollinated genotype, organic farming, betalain, nitrate, total sugar, phenolic compounds, total dry matter, antioxidant

## Abstract

The growing interest of consumers in healthy organic products has increased the attention to the organic production of beetroot. In this regard, six field experiments were conducted in 2017 and 2018 in three different locations under the specific conditions of organic agriculture, and fifteen beetroot genotypes, including one F1 hybrid as a commercial control and one breeding line, were compared regarding the content of the total dry matter, total soluble sugar, nitrate, betalain, and total phenolic compounds in order to investigate the genetic potential of new and existing open-pollinated genotypes of beetroot regarding the content of their bioactive compounds. The results of this study indicated a significant impact of genotype (*p* < 0.05) on all measured compounds. Furthermore, results revealed a significant influence of the interactions of location × year (*p* < 0.05) on the beetroot composition, and, thus, the role of environmental conditions for the formation of tested compounds. The total dry matter content (TDMC) of beetroots varied between 14.12% and 17.50%. The genotype ‘Nochowski’, which possessed the highest total soluble sugar content with 14.67 °Bx (Brix), was among the genotypes with the lowest nitrate content. On the contrary, the cylindrical-shaped genotype ‘Carillon RZ’ (Rijk Zwaan), indicated the lowest sugar content and the highest nitrate concentration. The amount of total phenolic compounds ranged between 352.46 ± 28.24 mg GAE 100 g^−1^ DW (milligrams of gallic acid equivalents per 100 g of dry weight) and 489.06 ± 28.24 mg GAE 100 g^−1^ DW for the red-colored genotypes which is correlated with the high antioxidant capacity of the investigated genotypes. Due to the specifics of the required content of bioactive compounds for various products, the selection of suitable genotypes should be aligned with the intended final utilization.

## 1. Introduction

Consumption of vegetables due to their various benefiting effects, such as the reduced risk of cardiovascular disease, cancer, and diabetes, is associated with human health promotion [1]. Due to the high amount of biologically active compounds, processing, and product consumption of beetroot (*Beta vulgaris* subsp. *vulgaris* L.), a vegetable from the Chenopodiaceae family, has gained attention in recent years [2,3].

Several studies noted beetroot as a vegetable with remarkable health-promoting phytochemicals [2,4]. Beetroot pigmentation can be categorized into two main sources; betalains composing of betanin, isobetanin, vulgaxanthin I, and vulgaxanthin II, and phenolic compounds, including flavonoids, phenolic acids, and other organic and inorganic acids [5]. The presence of betalains and phenolic compounds in beetroot has made this crop to be among the ten vegetables with the highest antioxidant activity [6]. The phytochemicals acting as antioxidants fight against free radicals [7], which generate cancer [8], heart, and kidney illnesses [9]. Furthermore, beetroot contains various minerals such as iron, zinc, phosphorous, potassium, calcium, sodium, magnesium, manganese, and copper [3], and vitamins, such as vitamin A, B6, and C (especially in the foliage). The presence of ascorbic acid and carotenoids in beetroot can further raise the total antioxidant capacity [5,10]. Another bioactive compound, which can be found in high levels in beetroot, is nitrate. Recently, the benefiting effects of nitrate on human health, including normalizing the blood pressure, protection against ischemia-reperfusion damage [3], an increase of muscle efficiency, and fatigue resistance [2], has drawn attention.

Considering the constantly increasing demand for organic products [11], different studies compared the quality of organic and conventional food products and reported lower amounts of harmful components and a higher amount of health-benefiting compounds in organically produced foods [12,13,14,15]. Furthermore, regarding the sensory quality, a better taste and smell were reported for red-colored beetroot grown organically [16]. In organic farming, the selection of the genotypes should comply with the principles of organic agriculture. Although organic farming is very critical of the use of F1 hybrid genotypes, farmers and gardeners tend to use F1 hybrids for commercial reasons. In comparison, the open-pollinated genotypes due to their benefiting features, including maintaining the plant integrity and self-reproductive ability [17,18], signify more advantages for the use in organic agriculture compared to F1 hybrids.

The objectives of the present study were to assess the content of bioactive compounds of 15 new and existing beetroot genotypes, consisting of 14 open-pollinated beetroot genotypes, which include one breeding line, and one F1 hybrid genotype, under the specific conditions of organic farming. Genotypes were tested in three pedoclimatic different locations to also test the impact of environmental conditions on the formation of bioactive compounds. Furthermore, this study intended to identify the open-pollinating genotypes of beetroot with outstanding chemical qualities similar to commercially used hybrids in order to introduce them to the organic industry and farmers as a substituent of hybrid genotypes.

## 2. Results and Discussion

### 2.1. Total Dry Matter Content

In accordance with the findings of this study, the TDMC of the 15 investigated beetroot genotypes varied between 14.12 ± 4.56% and 17.50 ± 4.56%. The results revealed that the genotypes Nochowski, Betina, and Cervena Kulata with 17.50 ± 4.56%, 17.12 ± 4.56%, and 16.41 ± 4.56%, respectively, contained the highest percentage of TDMC (Table 1). On the other hand, the cylindrical-shaped genotype, Carillon RZ with 14.12 ± 4.56%, Nobol with 14.66 ± 4.60%, and Akela RZ with 14.95 ± 4.56% indicated the lowest TDMC. The TDMC of 15 beetroot genotypes grown in Poland within 2009–2012 ranged between 13.5% to 15.3% [19], which were slightly lower than the range found in the current study. With respect to the outcomes of the analysis of variance (ANOVA), a significant impact of genotype (*p* = 0.0029) and the interactions of year × location (*p* < 0.0001) have been noted in the TDMC (Table 1). The assessment of the effect of cultivation method, genotype, and year on the total dry matter of two beetroot genotypes (cv. Czerwona Kula and cv. Opolski) grown in Poland, showed a content of 15.1% and 19.64% for cv. Czerwona Kula under organic cultivation and 15.1% and 19.46% for cv. Opolski in 2008 and 2009, respectively. In comparison, the TDMC of beetroots grown conventionally was reported as 14.3% and 18.79% for the cv. Czerwona Kula, and 13.6% and 18.66% for the cv. Opolski in 2008 and 2009, respectively [20]. That not only reveals the differences in the TDMC between the trial year and the genotypes but also significantly higher values in the organic cultivation. The lower TDMC of both cultivars in 2008, was due to the abundant rainfall and cooler days in the growing period compared to the year 2009 [20]. In the current study, the extreme climate condition in Germany (low precipitation level and high temperature) in the second year of the field experiment, resulted in the remarkable impact of the experimental year and location on the TDMC. Concerning the cultivation system, Brandt and Mølgaard (2001) stated that due to mineral nitrogen fertilization in conventional production systems, which increases the yield and the water quantity of the plant cells, often raw materials contain more water which leads to diluted content of the nutrients [21]. Moreover, Hallmann and Rembiałkowska (2012) claimed that the higher TDMC found in sweet bell peppers results from the difference in the metabolism of organic and conventional plants [22]. Nevertheless, Sikora et al. (2008) stated no significant influence of cultivation method on the TDMC of two beetroot cultivars Czerwona Kula and Regulski Cylindryczny [23].

### 2.2. Total Soluble Sugar Content

Due to the importance of carbohydrates as one of the main sources of energy as well as the amount of sugar in the controlled diet of the diabetic patients [24], providing information on the sugar composition and content in vegetables has gained more attention. Furthermore, due to the significant influence of processing practices on different soluble sugars, a need for the information on sugar amount has gained increasing interest for the food processing industry in order to optimize the processing conditions. The sugar contained in beetroot is mainly sucrose with small amounts of glucose and fructose [11].

The total soluble sugar content of the beetroot genotypes in the current work varied between 11.19 ± 0.37 °Bx and 14.67 ± 0.37 °Bx belonged to the genotypes Carillon RZ and Nochowski, respectively, and were statistically different from each other (Table 1). Following Nochowski, the genotypes Cervena Kulata with 13.94 ± 0.37 °Bx and Betina with 13.88 ± 0.37 °Bx possessed the highest total soluble sugar content. The study of Szopińska and Gawęda (2013) stated that the total soluble sugar content of the cylindrical-shaped genotype Regulski Cylinder grown organically in Poland varied between 7.61% and 8.16% within three years [25]. In the current study, higher contents were noted for the cylindrical-shaped genotype, Carillon RZ. Moreover, the amount of the total soluble sugar of 15 beetroot genotypes grown in Poland within 2009–2012 varied between 5.18% to 8.62% [19], which were considerably lower than the range found in the current study.

The amount of sugar content can be impacted by various factors. Statistical analysis of data of the current study determined a significant influence of genotype (*p* = 0.0028) and interactions of year × location (*p* < 0.0001) on the content of total soluble sugar (Table 1). Furthermore, Bavec et al. (2010) investigated the impact of different production systems, namely conventional, integrated, organic, and biodynamic, on the sugar content of red beet. Despite the differences in the sugar content of red beet grown under different farming conditions, no statistically significate influence of farming system was determined [11]. Another influencing factor on the sugar content of beetroot is the applied nitrogen in the early stages of growth and nitrogen availability [3]. In the present study, at the trial station De Beersche Hoeve, in the first year, the field was fertilized with compost equivalent to 60 kg N ha^−1^ and in the second year, compost equivalent to 90 kg N ha^−1^ was incorporated into the soil. At the Horticulture station Heinze, in both experimental years, no fertilizer was applied to the field and at the research station Kleinhohenheim, in the first year the field was fertilized with Vinasse, equivalent to 8.3 kg N ha^−1^, one month after seeding and in the second year, no additional fertilizer was used due to the N fixation by the pre-crop clover grass.

Providing information on the sugar content of each genotype is of great importance for the food industry for developing beetroot products without exceeding the maximum daily consumption and for achieving the best quality after processing. One of the critical products is prepared beetroot juice which contains approximately 62.0–92.0 g/L sugar [2]. Considering the World Health Organization (WHO) recommended daily sugar consumption of 25–50 g for an adult with normal Body Mass Index (BMI), with consumption of 300–600 mL of processed beetroot juice, the limit of daily sugar consumption can be meet [2]. Moreover, for those beetroot products in which intensive thermal treatment should be applied, considering the possibility of the occurrence of the Maillard reaction, the sugar content of the selected genotype should be taken into account.

### 2.3. Nitrate Content

The three predominant sources of human nitrate intake reported as water, cured meat, and vegetables [26], in which the green leafy vegetables like spinach, beets, lettuce, and radishes are the major dietary examples [27]. Heretofore, nitrate amount in the vegetables was discussed as a critical topic. That was due to the point that reduction of nitrate to nitrite leads to methemoglobinemia, which its reaction with secondary amines may sequentially form carcinogenic *N*-nitrosamines [28]. Latterly, with the reveal of the remarkable physiological importance of nitric oxide, the significant contribution of the dietary nitrate for the formation of the nitric oxide was disclosed [29].

Recently, the potential beneficial effects of dietary nitrate, including reducing the risk of cardiovascular diseases, gastric cancer, and hypertension [26] draw a lot of attention. Several studies noted the possible positive effects of nitrate on muscle efficiency, oxygen transport in the blood, and muscle fatigue of athletes, especially endurance athletes such as professional cyclists or marathon runners [30], therefore, consumption of beetroot as a good source of nitrate has drawn a lot of interest [31,32]. Depending on the final product and purpose of use, in order to have the suitable nitrate content, the choice of the appropriate cultivar has been highly emphasized [33,34].

The nitrate content of the investigated beetroot genotypes in the present study ranged between 4593 ± 1212 mg kg^−1^ DW and 10,924 ± 1211 mg kg^−1^ DW. Among all 15 genotypes, the three highest nitrate content were noted for Carillon RZ, Nobol, and Bona with 10,924 ± 1211 mg kg^−1^ DW, 9447 ± 1210 mg kg^−1^ DW, and 9364 ± 1221 mg kg^−1^ DW, respectively, which were statistically non-significantly different from each other (Table 1). On the other hand, the three genotypes, namely, Nochowski, BoRu1, and Monty RZ F1 with 4593 ± 1212 mg kg^−1^ DW, 4597 ± 1211 mg kg^−1^ DW, and 5487 ± 1209 mg kg^−1^ DW, respectively, showed the lowest nitrate content (Table 1). The reported nitrate values in Table 1 were expressed on a dry weight basis. Most studies express the nitrate content on a fresh weight (FW) basis, wherefore total dry matter content should be taken into account to compare our results to the literature. The findings of the current study were in agreement with the reported mean nitrate value of beetroots from different European countries, which was 1379 mg kg^−1^ FW [35]. The range of nitrate content of beetroot in Thessaloniki, Greece was between 443–981 mg kg^−1^ FW [36]. In the study of Rubóczki et al. (2015) the nitrate content in ten different beetroot genotypes grown in Hungry determined by a spectrophotometric method ranged between around 700–850 mg kg^−1^ FW [37]. Kosson et al. (2011) noted the mean nitrate contents of 1338.5 mg kg^−1^ FW and 1737.3 mg kg^−1^ FW for two beetroot genotypes grown in Poland, in 2008 and 2009, respectively [20].

The level of nitrate uptake and accumulation in vegetable tissues can be influenced by three main factors: environmental factors, such as water availability and irradiance, genetic factors, and agricultural factors, such as the application of fertilizers, herbicides, and other nutrients [38]. In accordance with the statistical analysis, the nitrate content was significantly affected by genotype (*p* = 0.0191) and interactions of year × location (*p* = 0.0495) (Table 1). This is in agreement with the findings of Kosson et al. (2011) who noted the significant impact of variety and year on the accumulation of nitrate in two different beetroot genotypes [20]. Furthermore, the effect of cultivation method (organic and conventional) was investigated in the study of Kosson et al. (2011) and 33% lower nitrate accumulation in the beetroot grown organically was reported [20]. In the current work, all beetroots were grown under organic conditions. Moreover, Vasconcellos et al. (2016) assessed the differences in the nitrate content of four beetroot products, including juice, powder, chips, and cooked beetroot. It was revealed that beetroot juice with 12,252.90 mg kg^−1^ DW had significantly higher nitrate concentration compared to all other products, which indicated the range of 1649.66–2031.20 mg kg^−1^ DW [5]. Therefore, it can be concluded that the amount of nitrate in beetroot products can be affected by processing. According to the previous studies, the variability in the nitrate content may occur among cultivars of the same species [39,40] genotypes and plant tissues [33,41,42,43]. In our study, each analyzed sample was a homogenized mixture of three randomly selected beetroot plants of each genotype, which were replicated three times in the field. Consequently, the values demonstrated in Table 1, represent the mean nitrate content of all three replications of each genotype cultivated in three different locations within two years to evaluate the genetic potential in different environmental conditions. Thus, due to the combination of various factors affecting the nitrate content, to have a better evaluation of different beetroot genotypes, a closer look at a larger number of plants per genotype might be appropriate.

### 2.4. Betalain Content

Color is one of the important attributes indicating the aesthetic and quality and consequently the acceptance of food [44]. Recently, due to the point that consumers prefer natural pigments compared to the synthetic ones, focus on the use of carotenoids, anthocyanins, and chlorophylls as examples of plant-based colorants [45] have been increased. Due to the limited edible sources of betalains, these pigments have been less commonly used for food coloring than other natural pigments [46]. Betalains are water-soluble, nitrogenous pigments [46] which are divided into two main structural groups, red-violet betacyanins and yellow-orange betaxanthins [47]. Previous studies reported the high antioxidant capacity of betalains [48]. Furthermore, the other important roles of betalains in human health are their anti-cancer, antilipidemic, and antimicrobial activities [47].

**Figure 1:**
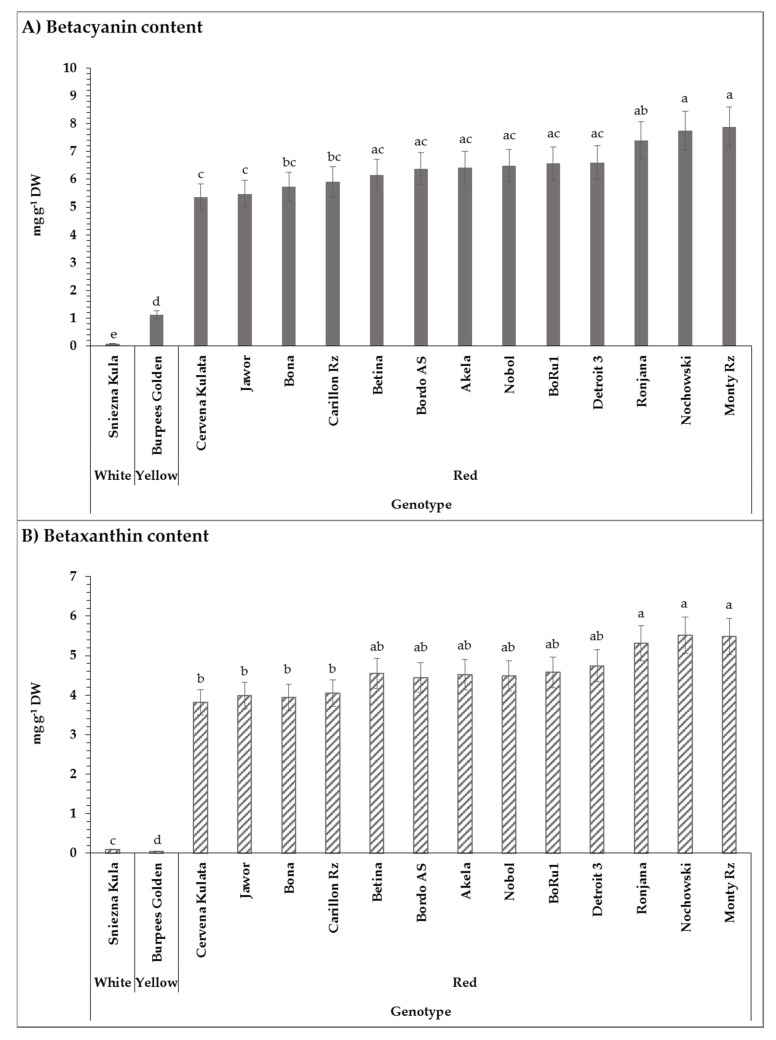
Mean values of (**A**) betacyanin content (mg g^−1^ DW) and (**B**) betaxanthin content (mg g^−1^ DW) of 15 different genotypes of beetroot grown in three research stations within the trial year 2017 and 2018. Means covered with at least one identical letter were not significantly different from each other.

In the present work, two main subgroups of betalain as representatives of the sample pigment content were quantified. Regarding betacyanin content, the studied red-colored genotypes ranged from 5.35 ± 0.49 mg g^−1^ DW to 7.89 ± 0.72 mg g^−1^ DW for the genotypes Cervena Kulata and Monty RZ F1, respectively (Figure 1). The white- and yellow-colored genotypes possessed the lowest betacyanin content with 0.08 ± 0.01 mg g^−1^ DW and 1.13 ± 0.14 mg g^−1^ DW, respectively, which were statistically different from each other (Figure 1). Following the F1 hybrid, Monty RZ, two genotypes Nochowski with 7.75 ± 0.70 mg g^−1^ DW and Ronjana with 7.39 ± 0.67 mg g^−1^ DW showed the highest betacyanin content. The betaxanthin content of the red-colored genotypes varied between 3.81 ± 0.32 mg g^−1^ DW and 5.51 ± 0.46 mg g^−1^ DW. The three highest betaxanthin content belonged to the genotypes Nochowski, Monty RZ F1, and Ronjana with 5.51 ± 0.46 mg g^−1^ DW, 5.49 ± 0.46 mg g^−1^ DW, and 5.32 ± 0.45 mg g^−1^ DW, respectively (Figure 1). The lowest betaxanthin contents were noted as 0.04 ± 0.01 mg g^−1^ DW and 0.09 ± 0.01 mg g^−1^ DW, belonging to the yellow-colored genotype, Burpees Golden, and the white-colored one, Sniezna Kula, respectively (Figure 1).

The results of ANOVA showed a significant effect of genotype (*p* < 0.0001) and interactions of year × location (*p* = 0.0277) on the betacyanin content. The effect of genotype (*p* < 0.0001) and interactions of year × location (*p* = 0.0069) was noted to be significant on the content of betaxanthin. Means and *p*-values are available in Table S1 in the supplementary material. The significant influence of genotype on betacyanin and betaxanthin content has been reported by Sawicki et al. (2016) [49]. Felczynski and Elkner (2008) stated the significant impact of weather conditions and consequently year of the experiment on the composition of the betalains in beetroot. Their study investigated the ratio of betanin to vulgaxanthin, which are respectively the main red and yellow pigments in beetroot, in two red beetroot cultivars, Chrobry and Nochowski, within two years and considerable differences have been noted [39], which is in agreement with the outcomes of the present study.

**Figure 2:**
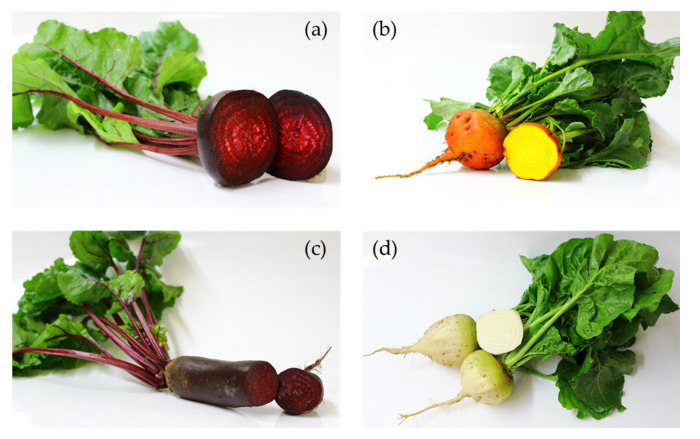
Presentation of four beetroot genotypes according to the beet shape and color (**a**) genotype Ronjana (red and round beet), (**b**) genotype Burpees Golden (yellow and round beet), (**c**) genotype Carillon RZ (red and cylindrical-shaped beet), and (**d**) genotype Sniezna Kula (white and round beet). Pictures are taken by Khadijeh Yasaminshirazi.

Kujala et al. (2000) stated that there is a different betalain distribution among the different parts of red beetroot [50]. The study reported higher betacyanin content in outer parts of red beetroot (cv. Little Ball), increasing in the order flesh, crown, and peel. Slatnar et al. (2015) studied the betacyanin and betaxanthin content in the peel and flesh of three different beetroot genotypes, including two red-colored and one yellow-colored genotype, which were grown in Slovenia, using the HPLC method. Regarding the two red-colored genotypes, the betacyanin content of 20.93 mg g^−1^ DW and 11.83 mg g^−1^ DW in the peel and 5.25 mg g^−1^ DW and 4.13 mg g^−1^ DW in the flesh of Pablo and Tanus, respectively, was measured [45]. The beetroot samples used in the current work mainly consisted of flesh with a small portion of the peel. Therefore, the slightly higher betacyanin and betaxanthin contents reported in this study (Figure 2) might be due to the different plant parts. Moreover, the betanin content of four red-colored beetroot genotypes measure by Kujala et al. (2002) ranged between 3.8–7.6 mg g^−1^ DW in the peel and 2.9–5.2 mg g^−1^ DW in the flesh. That for the vulgaxanthin content was reported as 1.4–4.3 mg g^−1^ DW and 1.5–4.0 mg g^−1^ DW in the peel and flesh, respectively [10]. The results of this study indicated a nearly similar range for the betacyanin content but higher betaxanthin values. Inconsistent to the previous findings, the study of Gasztonyi et al. (2001) presented a significantly lower range of betanin content of five red-colored beetroot genotypes with 0.41–0.50 g kg^−1^ (equivalent to mg g^−1^ in the current study) as well as lower range of vulgaxanthin I with 0.32–0.42 g kg^−1^ [51].

With respect to the betacyanin content of the yellow beetroot genotype, Boldor, Slatnar et al. (2015) stated 0.61 mg g^−1^ DW in the peel and 0.13 mg g^−1^ DW in the flesh. Likewise, for the betaxanthin content, the values of 4.71 mg g^−1^ DW and 0.22 mg g^−1^ DW in the peel and flesh, respectively, were reported [45]. The findings of our work regarding the yellow-colored genotype, Burpees Golden, indicated higher betacyanin content and remarkably lower betaxanthin value. Furthermore, the outcomes of Lee et al. (2014) demonstrated the betacyanin content of 0.114 mg g^−1^ FW and betaxanthin content of 0.187 mg g^−1^ FW for the mixed sample of peel and flesh of the yellow-colored beetroot, Burpees Golden, grown in Texas (the USA) [52].

Providing information on the betalain content of each beetroot genotype, due to the effect on the antioxidant activity as well as the color intensity, is important for the food industry in order to choose the suitable genotype for the final product such as for the use of beetroot in functional processed food or the use as the food colorant.

### 2.5. Total Phenolic Content

Phenolic compounds are plants’ secondary metabolites, which affect both sensorial attributes, such as flavor, taste and color, and functional properties, like antioxidant activity, of plant products [53,54]. Furthermore, phenolic compounds also contribute to plants’ growth, pigmentation, reproduction [55], and defense mechanisms under different stress conditions. Due to the free radicals scavenging activity, phenolic compounds demonstrate a high antioxidant effect. Antioxidant activity is associated with the prevention of cardiovascular diseases and cancer [56,57].

The total phenolic content ranged from 352.46 ± 28.24 mg GAE 100 g^−1^ DW to 489.06 ± 28.24 mg GAE 100 g^−1^ DW for the red-colored genotypes. The F1 hybrid genotype, Monty RZ, showed the highest total phenolic content followed by Cervena Kulata and Nochowski (Table 1). On the other hand, the genotypes Carillon RZ, BoRu1, and Betina with 352.46 ± 28.24 mg GAE 100 g^−1^ DW, 382.73 ± 28.24 mg GAE 100 g^−1^ DW, and 393.94 ± 28.24 mg GAE 100 g^−1^ DW, respectively, indicated the lowest total phenolic content among the studied red-colored genotypes. The yellow-colored genotype, Burpees Golden, and the white-colored genotype, Sniezna Kula, with 172.89 ± 28.78 mg GAE 100 g^−1^ DW and 216.09 ± 29.87 mg GAE 100 g^−1^ DW, respectively, demonstrated significantly lower total phenolic content compared to the red genotypes (Table 1). In the study of Rubóczki et al. (2015), the content of total polyphenols in ten different beetroot genotypes grown in Hungary was determined using the Folin–Ciocalteu method and varied between 45.47 mg GAE/100 g and 83.37 mg GAE/100 g [37]. Values were considerably lower than the values measured in our study. Furthermore, Ninfali and Angelino (2013) reported total phenols of 1.77 mg GAE g^−1^ DW (equivalent to 177 mg GAE 100 g^−1^ DW) [58], which was lower than the values found in the current study. Kovarovič et al. (2017) investigated the total phenolic compounds of four beetroot varieties in the Czech Republic and noted the range of 368.75 ± 5.14 mg kg^−1^ (equivalent to 36.87 mg 100 g^−1^ DW) to 887.75 ± 7.73 mg kg^−1^ (equivalent to 88.77 mg 100 g^−1^ DW) belonging to the variety Chioggia (white-colored with red strips) and Cylindra, respectively [59]. Kavalcova’ et al. (2015) stated a total polyphenols’ range of 820.10 mg kg^−1^ to 1280.56 mg kg^−1^ (equivalent to 82.01–128.05 mg 100 g^−1^ DW) [60]. The investigated genotypes in our study exhibited promising results regarding the amount of total phenolic compounds.

The outcomes of ANOVA indicated a significant impact of genotype (*p* = 0.0002) and the interactions of year × location (*p* = 0.0114) (Table 1). The total phenol content in fresh fruits and vegetables can be affected by different biotic and abiotic factors. Genotype, environmental conditions such as climate and soil, and plant maturity and ontogeny play the most important role in the quantity of phenolic compounds [55]. It was reported that the level of chemical fertilizers, which are used in conventional agricultural practices, can lead to the disturbance in the natural production of phenolic compounds [61]. In this regard, Carrillo et al. (2019) investigated the impact of the production system on the content of total phenols in six different beetroot genotypes. It was reported that the total polyphenol content of the organic beetroots was significantly higher than the conventional ones, although the difference depended on the cultivar [62]. Nevertheless, the study of Straus et al. (2012) demonstrated a higher total phenolic content in beetroots grown under organic condition compared to the conventional ones, however, the difference was not significant [63].

Similar to the betalain content in beetroot, several studies noted a higher total phenolic content in the peel than in the flesh [50,64]. Based on the point that the analyzed samples in the current work mainly consisted of flesh with a small proportion of peel, the differences between the values reported in other studies and the outcomes of this study might be due to the variation in the plant part. Additionally, differences in the measurement methods [65] (e.g., HPLC, capillary zone electrophoresis, and Folin–Ciocalteu), as well as the extraction of the phenolic compounds techniques [66,67,68] in various studies, may lead to a variation in the reported values.

## 3. Methods and Materials

### 3.1. Plant Materials and Sample Preparation

The analyzed beetroot samples were obtained from six field experiments, which were carried out in two years, 2017 and 2018, at three different locations. Locations consisted of two on-farm breeding locations of Kultursaat e.V.: De Beersche Hoeve (Oostelbeers, Netherlands), Horticulture station Heinze (Bingenheim, Hessia, Germany), and at the research station for organic farming Kleinhohenheim (University of Hohenheim, Stuttgart, Baden-Wuerttemberg, Germany). All beetroots were grown organically. In 2017, on each on-farm breeding location 30, and in Kleinhohenheim, 40 genotypes were cultivated in three field replicates. In 2018, 16 genotypes were investigated on each on-farm breeding location while 36 genotypes were assessed in Kleinhohenheim. Data from all genotypes were analyzed together. However, the presented results were limited to the 15 genotypes, which occurred in all location-by-year combinations. The 15 investigated genotypes can be listed as 13 red-colored genotypes, Akela RZ, Betina, Bona, Bordo, BoRu1, Carillon RZ, Cervena Kulata, Detroit 3, Jawor, Monty RZ F1, Nobol, Nochowski, Ronjana, one white genotype, Sniezna Kula, and a yellow genotype, Burpees Golden. Figure 2 illustrates four beetroot genotypes representing different beet shapes and colors, which were investigated in this study. The detailed information on the seeds’ origin, experimental design, and the locations can be found in Yasaminshirazi et al. (2020) [69].

Samples were prepared from three randomly selected beetroots per plot, which fulfilled the criteria for marketability (beet diameter between 5 and 13 cm and without any deformation and notable damages). Beetroots were washed directly after the harvest. After cutting out the root tail and the leaves-growth-base, a sectional cut of the beet including the peel were chopped into small pieces and thoroughly mixed to have a homogenous sample. The chopped pieces were collected in a closed plastic flask and immediately were frozen using liquid nitrogen. Afterward, the samples were lyophilized using the Dieter Piatkowski–Forschungsgeraete freeze-dryer (Munich, Germany). The dried samples were ground by a laboratory knife mill (Retsch GM 200, Haan, Germany) until they reached a fine powder texture. The powder was collected and remained in a closed small plastic flask in a cool dark box until the day the chemical analyses were carried out.

### 3.2. Chemicals and Reagents

The Folin–Ciocalteu reagent and gallic acid were purchased from Merck (Darmstadt, Germany). Na_2_CO_3_ was provided by AppliChem GmbH (Darmstadt, Germany). Methanol and ethanol were purchased from Carl Roth GmbH (Karlsruhe, Germany) and Th. Geyer (Renningen, Germany), respectively. Regarding the nitrate measurement, sulfanilamide was purchased from AppliChem GmbH (Darmstadt, Germany). Ammonium chloride and hydrochloric acid were provided by Th. Geyer (Renningen, Germany). Sodium nitrite and ammonia solution 25% from Merck (Darmstadt, Germany) and N-(1-naphthyl)-ethylene diamine dihydrochloride from Carl Roth GmbH (Karlsruhe, Germany) were used.

### 3.3. Total Dry Matter Content

The weight of chopped beetroot samples (*i*) was measured before and after freeze-drying and the total dry matter content (TDMC) was calculated according to Equation (1):

(1) $${\mathrm{TDMC}}_{i}\;\left[\%\right]=\left(\frac{weight\;after\;drying_{i}}{weight\;before\;drying_{i}}\right)\times 100$$

### 3.4. Total Soluble Sugar Content

The determination of total soluble sugar content was carried out using a digital handheld refractometer (Kruess, Germany). One to two drops of freshly pressed beetroot were used to determine the degree of Brix, which is equivalent to the percentage of total soluble sugar content. Each sample was measured in duplicate and a mean value was calculated directly.

### 3.5. Quantitation of Nitrate Content

Approximately 0.5 g of each dried beetroot sample was weighed and added in a 100 mL volumetric flask and was filled up with distilled water until the final volume adjusted to 100 mL and was shaken shortly, manually. The volumetric flask was placed in an ultrasonic water bath at 80 °C for 10 min. After the flask was cooled down, the supernatant was filtered using filter paper. The nitrate content quantitation was conducted by flow injection analysis method (FIA) [27] using FIASTAR 5000 (FOSS Analytical AB, Sweden).

### 3.6. Betalain Content

#### 3.6.1. Extraction of Betalains

Approximately 0.04 g of each dried sample was weighed (Precisa 125 A, Dietikon, Switzerland) and added to 30 mL of 50% (*v/v*) ethanol in a 50 mL falcon tube. The mixture was shaken for two hours with 100 rounds per minute (rpm). Afterward, the obtained extracts were centrifuged (Centrifuge 5810 R, Eppendorf AG, Hamburg, Germany) with 4000 rpm and at 20 °C for 10 min.

#### 3.6.2. Betalains Content Determination

Quantification of two main subgroups of betalains, betacyanin, and betaxanthin, was determined spectrophotometrically using a UV/Visible Spectrophotometer (Ultrospec 3100 Pro, Amersham Bioscience, Buckinghamshire, UK). The absorption of betacyanins at 538 nm and betaxanthins at 480 nm were measured and their concentrations were calculated based on Koubaier et al. (2014) and Sawicki et al. (2016) [49,70].

### 3.7. Total Phenolic Content

#### 3.7.1. Extraction of Total Phenolic Compounds

Approximately 0.5 g of each dried beetroot sample was weighed and collected in a 15 mL falcon tube where 10 mL of methanol was added. The mixture was shaken for 30 min and afterward centrifuged (Centrifuge 5810 R, Eppendorf AG, Hamburg, Germany) at 4000 rpm at 20 °C for 20 min in order to separate the solid phase from the supernatant. These extracts were utilized for the measurement of the total phenolic content.

#### 3.7.2. Total Phenolic Content Quantification

The total phenolic content measurement was carried out following the methodology of Folin–Ciocalteu [71]. Summarily, 0.6 mL of the extracted sample was added to 60 mL of distilled water in a 100 mL volumetric flask. Then, 5 mL of Folin–Ciocalteu reagent was added to the flask. After two to six minutes, 25 mL of sodium carbonate solution 15% was added to the mixture, and the final volume was adjusted to 100 mL with distilled water. After two hours of incubation at room temperature, absorbance at 760 nm was determined by a UV/Visible Spectrophotometer (Ultrospec 3100 Pro, Amersham Bioscience, Buckinghamshire, UK). To draw a calibration curve, six standard solutions consisting of 0.03 g L^−1^, 0.12 g L^−1^, 0.24 g L^−1^, 0.48 g L^−1^, 0.9 g L^−1^, and 1.5 g L^−1^ gallic acid in distilled water were used. Lastly, the total phenolic content was stated as mg GAE 100 g^−1^ DW (milligrams of gallic acid equivalents per 100 g of dry weight).

### 3.8. Statistical Analysis

According to the design of the experiments, the data were analyzed by the linear mixed model used in Yasaminshirazi et al. (2020) [69] on agronomic traits from the same six experiments:

(2) $$y_{ijklmn}=\mu +b_{ljk}+r_{mjk}+c_{njk}+a_{j}+l_{k}+\tau_{i}+{\left(al\right)}_{jk}+{\left(\tau a\right)}_{ij}+{\left(\tau l\right)}_{ik}+{\left(\tau al\right)}_{ijk}+e_{ijklmn},\;$$

where $a_{j}$ and $l_{k}$ are the fixed effects of the *j*-th year and the *k*-th location. $b_{ljk}$, $r_{mjk}$, and $c_{njk}$ are the random effect of the *l*-th block, *m*-th row and *n*-th column within a year-by-location combination. Note that block effects as well as row and column effects were only fitted if blocks existed and a row-column design was used, respectively. Dummy variables were used to block out these effects for all other experiments but were not mentioned in (2) to simplify the description. $\tau_{i}$ is the fixed effect of the *i*-th genotype. ${\left(al\right)}_{jk}$ is the fixed interaction effect of the *j*-th year and the *k*-th location. ${\left(\tau a\right)}_{ij}$, ${\left(\tau l\right)}_{ik}$, and ${\left(\tau al\right)}_{ijk}$ are the random interaction effects between the corresponding main effects. $e_{ijklmn}$ is the error of $y_{ijklmn}$ with year-by-location specific error variance. For the trait total phenolic content, a group-by-location-specific error variance was assumed, where a group refers to beetroots of the same color (red, yellow, white). For betacyanin and betaxanthin, a group-by-year-specific error variance was found to fit well. The Akaike Information Criterion (AIC) was used to select the best error variance structure [72]. The assumptions of normality of residuals and homogeneity of residual variances (in addition to the heterogeneity specified in the model) were checked graphically. For the compounds betacyanin and betaxanthin, a logarithmic transformation of the data was used before analysis. Estimated means were back-transformed afterward for presentation purposes only. These means represent medians on the original scale. Standard errors were back-transformed using the delta method. Depending on the significance of fixed effects, mean comparisons were done for the highest significant interaction term of all factors using Fisher´s Least Significant Difference (LSD) test [73]. Means (or medians in case of back-transformed values) and their (approximate) standard error were presented. A letter display was created using the statistical software SAS version 9.4 (SAS Institute, Cary, North Carolina, United States) macro %mult [73] to present results from multiple comparisons.

## 4. Conclusions

Significant differences were found in the content of selected compounds between the assessed open-pollinated beetroot genotypes. This outcome is essential for the selection of genotypes and is of great importance for farmers for having new possibilities for cultivation in organic farming, for the industry for the production and development of beetroot products with desirable characteristics, and for meeting consumers’ expectations of health-promoting food products. Furthermore, with the significant impact of the interactions of year × location on all tested compounds, it can be concluded that the abiotic factors influence the biochemical composition of beetroot. Out of the 15 tested genotypes the open-pollinated genotype Nochowski with comparable total phenolic compounds and betacyanin content to F1 hybrid genotype, Monty F1 RZ, indicated a high antioxidant capacity, which makes it a potential genotype for value-added food products. Moreover, this genotype may serve as an option for juice production due to its high sugar and low nitrate content. According to the importance of taste on customers’ acceptability, further studies in association with the sensory quality of the genotypes as well as other flavor-relevant components, such as geosmin, are required.

## Figures and Tables

**Table 1:** Mean values and ANOVA of results of the content of nitrate (mg kg^−1^ dry weight (DW)), total soluble sugar (°Bx), total phenolic (mg gallic acid (GAE) 100 g^−1^ DW), and total dry matter (%) of 15 different genotypes of beetroot grown in three research stations within the trial year 2017 and 2018. Results represent the mean values ± standard error. Means followed by at least one identical letter were not significantly different from each other.

Genotype	Nitrate (mg kg^−1^ DW)	Total Soluble Sugar (°Bx)	Total Phenolic Content (mg GAE 100 g^−1^ DW)	Total Dry Matter Content (%)
Akela RZ	8865 ^a,c^ ± 1209	11.94 ^c,e^ ± 0.37	402.55 ^b,c^ ± 28.24	14.95 ^d,e,f^ ± 4.56
Betina	5817 ^b,c,d^ ± 1210	13.88 ^a,b^ ± 0.37	393.94 ^b,c^ ± 28.24	17.12 ^a,b^ ± 4.56
Bona	9364 ^a,b^ ± 1221	12.39 ^c,d^ ± 0.37	395.55 ^b,c^ ± 28.24	15.44 ^c,e,f^ ± 4.56
Bordo	7023 ^b,c,d^ ± 1210	12.80 ^b,c^ ± 0.37	420.29 ^a,c^ ± 28.24	16.21 ^a,b,c,d^ ± 4.56
BoRu1	4597 ^d^ ± 1211	12.47 ^c^ ± 0.37	382.73 ^c^ ± 28.24	15.47 ^c,e,f^ ± 4.56
Burpees Golden	7566 ^a,d^ ± 1211	11.94 ^c,e^ ± 0.37	172.89 ^d^ ± 28.78	15.98 ^b,e^ ± 4.56
Carillon RZ	10924 ^a^ ± 1211	11.19 ^e^ ± 0.37	352.46 ^c^ ± 28.24	14.12 ^f^ ± 4.56
Cervena Kulata	5768 ^b,c,d^ ± 1228	13.94 ^a,b^ ± 0.37	476.84 ^a,b^ ± 28.24	16.41 ^a,b,c,d^ ± 4.56
Detroit 3	7682 ^a,d^ ± 1211	11.99 ^ce^ ± 0.37	402.61 ^b,c^ ± 28.24	15.06 ^c,e,f^ ± 4.56
Jawor	7855 ^a,d^ ± 1211	12.60 ^c^ ± 0.37	415.82 ^a,c^ ± 28.24	15.55 ^c,e^ ± 4.56
Monty RZ F1	5487 ^c,d^ ± 1209	12.33 ^c,e^ ± 0.37	489.06 ^a^ ± 28.24	15.41 ^c,e,f^ ± 4.56
Nobol	9447 ^a,b^ ± 1210	11.28 ^d,e^ ± 0.37	401.23 ^b,c^ ± 28.24	14.66 ^e,f^ ± 4.60
Nochowski	4593 ^d^ ± 1212	14.67 ^a^ ± 0.37	432.90 ^a,c^ ± 28.24	17.50 ^a^ ± 4.56
Ronjana	8595 ^a.c^ ± 1210	12.80 ^b,c^ ± 0.37	405.32 ^a,c^ ± 28.24	15.34 ^c,e,f^ ± 4.56
Sniezna Kula	7427 ^a,d^ ± 1211	11.91 ^c,e^ ± 0.37	216.09 ^d^ ± 29.87	16.05 ^b,e^ ± 4.56
**Factor**	***p*-Value of the F-Test of the Corresponding Factor**			
Location	<0.0001	0.0008	n.s. ^1^	0.0013
Year	n.s.	<0.0001	<0.0001	<0.0001
Genotype	0.0191	0.0028	0.0002	0.0029
Location × year	0.0495	<0.0001	0.0114	<0.0001

^1^ not significant.

## Supplementary Materials

The following are available online, Table S1: Mean values and ANOVA of results of betacyanin (mg g^−1^ DW) and betaxanthin (mg g^−1^ DW) content of 15 different genotypes of beetroot grown in three research stations within the trial year 2017 and 2018.
