# Supplementary material for: Bioactive Compounds and Total Sugar Contents of Different Open-Pollinated Beetroot Genotypes Grown Organically
Source: Molecules. 2020 Oct 22;25(21):4884. doi: 10.3390/molecules25214884 (PMC7660082; doi:10.3390/molecules25214884)
Supplement: Supplementary file 1 [file molecules-25-04884-s001.pdf]

Table S1. Mean values and ANOVA of results of betacyanin (mg g^{-1} DW) and betaxanthin (mg g^{-1} DW) content of 15 different genotypes of beetroot grown in three research stations within the trial year 2017 and 2018. Results represent the mean values ± standard error. Means followed by at least one identical letter were not significantly different from each other

**Genotype**	**Betacyanin (mg g^{-1} DW)**	**Betaxanthin (mg g^{-1} DW)**
Akela	6.42 ^{ac} ± 0.58	4.52 ^{ab} ± 0.38
Betina	6.16 ^{ac} ± 0.56	4.54 ^{ab} ± 0.38
Bona	5.73 ^{bc} ± 0.52	3.95 ^{b} ± 0.33
Bordo AS	6.39 ^{ac} ± 0.58	4.45 ^{ab} ± 0.37
BoRu1	6.57 ^{ac} ± 0.60	4.57 ^{ab} ± 0.38
Burpees Golden	1.13 ^{d} ± 0.14	0.04 ^{d} ± 0.01
Carillon RZ	5.91 ^{bc} ± 0.54	4.05 ^{b} ± 0.34
Cervena Kulata	5.35 ^{c} ± 0.49	3.81 ^{b} ± 0.32
Detroit 3	6.60 ^{ac} ± 0.60	4.74 ^{ab} ± 0.40
Jawor	5.46 ^{c} ± 0.50	3.99 ^{b} ± 0.34
Monty RZ F1	7.89 ^{a} ± 0.72	5.49 ^{a} ± 0.46
Nobol	6.49 ^{ac} ± 0.59	4.49 ^{ab} ± 0.38
Nochowski	7.75 ^{a} ± 0.70	5.51 ^{a} ± 0.46
Ronjana	7.39 ^{ab} ± 0.67	5.32 ^{a} ± 0.45
Sniezna Kula	0.08 ^{e} ± 0.01	0.09 ^{c} ± 0.01
**Factor**	***p*-value of the F-test of the corresponding factor**	
Location	0.0056	0.0009
Year	n.s.	0.0045
Genotype	<0.0001	<0.0001
Location × year	0.0277	0.0069
